# Visible injury patterns in relation to reported sexual acts after sexual assault: a retrospective cohort study

**DOI:** 10.1007/s00414-026-03941-8

**Published:** 2026-08-01

**Authors:** Alice Buzzelli, Julia Babigian, Valentina Bugelli, Lars Oesterhelweg, Alberto Amadasi

**Affiliations:** 1 Department of Legal Medicine, University of Parma, Viale Antonio Gramsci 14, Parma, 43100 Italy; 2 Institute of Legal Medicine and Forensic Sciences, Charité-Universitätsmedizin Berlin, corporate member of Freie Universität Berlin and Humboldt-Universität zu Berlin, Institute of Legal Medicine and Forensic Sciences, Turmstrasse 21, Berlin, 10559 Germany; 3 Berlin Institute of Health at Charité – Universitätsmedizin Berlin, BIH Biomedical Innovation Academy, BIH Charité Junior Clinician Scientist Program, Charitéplatz 1, Berlin, 10117 Germany

**Keywords:** Sexual assault, Forensic medicine, Genital injuries, Anogenital injuries, Extragenital injuries, Visible injuries, Anatomical correspondence

## Abstract

Sexual assault may result in visible anogenital and extragenital injuries, but the absence of such findings remains a major challenge in forensic interpretation. This retrospective forensic outpatient study analyzed 306 adults examined after reported sexual assault at the Violence Protection Ambulatory of Charité – Universitätsmedizin Berlin between January 2016 and December 2023. Demographic, assault-related, and injury-related variables were extracted from finalized forensic examination reports. The primary aim was to assess the frequency of predefined anatomically or regionally corresponding visible injuries in relation to reported oral, vaginal, and anal sexual acts. Extragenital injuries were documented in 216/306 cases (70.6%), whereas anogenital injuries were documented in 62/306 cases (20.3%). Among 256 individuals reporting at least one oral, vaginal, or anal sexual act, predefined corresponding visible injuries were present in 72 cases (28.1%, 95% CI 23.0–33.9) and absent in 184 cases (71.9%). Corresponding anogenital injuries were uncommon after reported vaginal acts and were not significantly associated with reported vaginal acts or penile-vaginal penetration. Reported anal sexual violence and oral sexual violence were significantly associated with anal and facial injuries, respectively; however, corresponding injuries were still absent in most cases. In multivariable logistic regression, the number of reported sexual acts was the only variable independently associated with absence of corresponding visible injury. These findings demonstrate the limited sensitivity of visible injury documentation after reported sexual assault. The presence of a corresponding injury may support the assessment of consistency with the reported history, but the absence of visible injury must not be interpreted as excluding the reported assault.

**Supplementary Information:**

The online version contains supplementary material available at https://doi.org/10.1007/s00414-026-03941-8.

## Introduction

Sexual assault is a major public health and medico-legal issue worldwide. Data from the European Institute for Gender Equality indicate that violence against women, including physical and/or sexual violence, remains highly prevalent and is frequently associated with adverse health consequences [1]. In this context, forensic medical examination plays a central role in documenting injuries, assessing the consistency of findings with the reported history, and providing objective medico-legal documentation for potential legal proceedings. However, the interpretation of injuries after reported sexual assault remains one of the most challenging areas of forensic medical practice [2]. Genital and anogenital injuries have received particular attention because their documentation may influence medico-legal assessment and criminal justice outcomes [3]. At the same time, previous studies comparing consensual and non-consensual sexual intercourse have demonstrated substantial overlap in genital injury patterns, limiting the diagnostic value of genital findings when interpreted in isolation [4, 5]. Accordingly, both the presence and the absence of visible injuries must be interpreted cautiously and in relation to the reported assault dynamics, timing of examination, and contextual case information. Retrospective studies from different geographic and sociocultural settings have described the epidemiological and medico-legal characteristics of sexual assault cases, including affected individuals’ characteristics, reported assault circumstances, and the prevalence of genital, anal, and extragenital injuries [6–16]. These studies consistently show that physical injuries are variable and that the absence of visible injury is common, even after reported penetrative sexual assault. Despite this body of literature, less attention has been paid to the anatomical correspondence between reported sexual acts and documented visible injuries. In routine forensic practice, this distinction is important: an injury may be present but anatomically unrelated to the reported sexual act, while a reported act may leave no visible corresponding injury. A systematic evaluation of such correspondence may therefore help to clarify the evidentiary limits of visible injury documentation after reported sexual assault. The aim of the present study was to describe genital, anogenital, and extragenital injury patterns in adults examined after reported sexual assault at a forensic outpatient clinic in Berlin and to assess the frequency of predefined anatomically or regionally corresponding visible injuries in relation to reported oral, vaginal, and anal sexual acts. As a secondary objective, forensic interpretation categories assigned during medico-legal assessment were descriptively analyzed.

## Materials and methods

### General characteristics of the study

The forensic specialists at the Violence Protection Ambulatory (GSA) at Charité - Universitätsmedizin Berlin conduct patient examinations by appointment on-site and, in cases of sexualised violence (SG), on behalf of the police within the Charité emergency departments. Additionally, a confidential forensic service (VSS) operates independently of police involvement. A mobile service (MD) is also available to examine patients in Berlin hospitals and social institutions, including women’s shelters. The Violence Protection Ambulatory operates during regular office hours. Outside these hours, affected individuals requiring urgent medical care are assessed in the Charité emergency departments by the appropriate clinical specialists. Forensic medical examinations by the Violence Protection Ambulatory are performed by forensic physicians during office hours. Consequently, some individuals included in the present study initially received emergency clinical care overnight and subsequently underwent forensic examination at the Violence Protection Ambulatory on the following working day. Forensic examinations included in the present study were performed according to the routine organization of the Violence Protection Ambulatory. Extragenital injuries were assessed by forensic physicians through systematic visual inspection under daylight and artificial light, whereas anogenital examinations were performed by gynecologists using standard gynecological examination techniques, including visual inspection, colposcopy, and speculum examination when clinically indicated. The findings of these examinations were documented in the finalized forensic reports used for the present study. This retrospective cohort study included all adult individuals (aged 18 years and older) who underwent forensic medical examination at the GSA between January 2016 and December 2023, following reported sexual assault. Inclusion criteria were age ≥ 18 years and forensic medical examination after reported sexual assault involving, or potentially involving, oral, vaginal, or anal sexual contact. Cases were also eligible if the affected individual was uncertain about the specific assault dynamics. Cases were included irrespective of whether visible injuries were documented. Cases were excluded if the affected individual was younger than 18 years, if the available documentation did not refer to a reported sexual assault within the scope of the present study, or if the forensic documentation was insufficient for extraction of the variables required for analysis. Standardized forensic documentation forms and final forensic examination reports served as the primary data sources. The reports had been completed in routine forensic practice on the basis of written examination findings and corresponding photographic documentation. For the present retrospective study, the finalized forensic reports were systematically reviewed, and predefined variables were extracted into a Microsoft Excel database. Available photographic documentation was used only to verify documented findings during data extraction where necessary; no independent re-assessment of injury findings was performed for the purpose of this study. Extracted demographic and case-related variables included sex of the affected individual, sex of the reported offender, age, height, weight, body mass index, documented physical disability, date of the reported assault, date of forensic examination, and the time interval between the reported assault and examination in hours. The relationship between the affected individual and the reported offender was categorized as current or former intimate partner, known person, or unknown person. Information on the number of reported offenders, alcohol and drug involvement reported by the affected individual, self-inflicted injuries, and reported non-fatal strangulation was also collected. Extragenital injuries were coded according to their presence, anatomical location, and morphological type. Anatomical locations included the neck, head, face, thorax, back, abdomen, upper arm, forearm, hands, and legs. Injury types included petechiae, abrasions, hematomas, bite marks, lacerated wounds, sharp-force injuries, and fractures. The severity of extragenital injuries was graded using the Clinical Injury Extent Score (CIES), as previously applied in sexual assault cohorts [2, 3]. The highest applicable CIES category par case was used for analysis. The CIES categories are provided in Supplementary Table S3. In addition to anatomical injury locations and morphological injury types, forensic interpretation categories documented in the original forensic records were extracted descriptively. These categories reflected the medico-legal interpretation of injury morphology, anatomical location, and contextual case information, and were not treated as independent anatomical injury outcomes. Categories included blunt force by blows or kicks, blunt force by objects, kissing or sucking injuries, defensive injuries, fixation injuries, strangulation marks, undressing injuries, spread injuries, and abutment injuries. Anogenital injuries were coded according to their presence, anatomical location, and morphological type. Anatomical locations included the labia majora, labia minora, clitoris, vestibulum, posterior fourchette, navicular fossa, hymen, anterior vagina/fornix, perineal region, anus, and penis. Morphological injury types included erythema, edema, hematoma, abrasion, and laceration. Anogenital injuries were coded as present or absent; no severity grading was applied. Reported sexual acts were categorized as oral sexual violence, penile-vaginal penetration, digital-vaginal penetration, vaginal penetration with objects, penile-anal penetration, digital-anal penetration, anal penetration with objects, or unknown/uncertain sexual dynamics. Categories were not mutually exclusive, and more than one category could be assigned if multiple acts were reported. Anatomical correspondence was assessed using predefined combinations of reported sexual acts and documented visible injury locations. For reported vaginal acts, any documented anogenital injury was considered anatomically corresponding. For reported anal acts, documented anal injury was considered anatomically corresponding. For reported oral sexual violence, documented facial injury was considered regionally corresponding. The composite endpoint “any anatomically corresponding injury” was defined as the presence of at least one corresponding visible injury among individuals reporting at least one oral, vaginal, or anal sexual act. Because reported sexual acts were not mutually exclusive, individuals could contribute to more than one act-specific analysis.

### Statistical analysis

Statistical analyses were performed using Microsoft^®^ Excel^®^ for Microsoft 365 MSO (Version 2505 Build 16.0.18827.20102) and IBM SPSS Statistics Version 30.0.0.0 (117). Descriptive statistics were used to summarize demographic, assault-related, and injury-related variables. Continuous variables are presented as mean ± standard deviation and median with interquartile range, as appropriate. Categorical variables are presented as absolute numbers and percentages. Percentages were calculated using the total study population as the denominator unless otherwise specified. For analyses restricted to individuals with at least one reported oral, vaginal, or anal sexual act, the respective subgroup size was used as the denominator. Missing data were not imputed; analyses were performed using available cases, and denominators are reported where they differ from the total cohort. Associations between reported sexual acts and predefined anatomically corresponding visible injuries were assessed using chi-square tests or Fisher’s exact tests, as appropriate. Continuous and ordinal variables were compared between groups using Mann–Whitney U tests. 95% confidence intervals were calculated for proportions. To evaluate factors associated with the absence of anatomically corresponding visible injuries, univariable analyses were first performed for demographic and assault-related variables. Variables of forensic or clinical relevance were then entered into a multivariable binary logistic regression model with absence of anatomically corresponding injury as the dependent variable. The model included age, time to examination, alcohol involvement, drug involvement, and number of reported sexual acts. Odds ratios (ORs) with 95% confidence intervals were reported. Model fit was assessed using the likelihood-ratio chi-square test, Nagelkerke’s R², and the Hosmer–Lemeshow goodness-of-fit test. All statistical tests were two-sided, and p-values < 0.05 were considered statistically significant. Given the retrospective and exploratory nature of the study, no correction for multiple testing was applied.

## Results

### Study population and documented injury characteristics

A total of 306 adult individuals examined after reported sexual assault were included in the analysis. The cohort was predominantly female (288/306, 94.1%); 15 individuals were male (4.9%) and three were documented as diverse (1.0%). The mean age was 30.0 years (SD 10.3), with a median age of 27.5 years (IQR 22–36). The median time interval between the reported incident and forensic examination was 24 h (IQR 24–48), with a mean interval of 38.5 h (SD 30.7). Baseline demographic, assault-related, and documented injury characteristics are summarized in Table 1. Most reported offenders were male (298/306, 97.4%). One offender was reported in 257 cases (84.0%), whereas two or more offenders were reported in 38 cases (12.4%). The reported offender was known to the affected individual in 159 cases (52.0%), unknown in 105 cases (34.3%), and a current or former intimate partner in 42 cases (13.7%). Reported alcohol involvement was documented in 150 cases (49.0%), and reported drug involvement in 51 cases (16.7%); in 26 cases (8.5%), drug involvement was unknown or uncertain to the affected individual. At least one relevant oral, vaginal, or anal sexual act was reported in 256 cases (83.7%). Vaginal acts were the most frequently reported modality (226/306, 73.9%), including penile-vaginal penetration in 190 cases (62.1%) and digital-vaginal penetration in 85 cases (27.8%). Anal acts were reported in 67 cases (21.9%), and oral sexual violence in 64 cases (20.9%). In 49 cases (16.0%), the specific type of sexual violence was unknown or uncertain to the affected individual. Extragenital injuries were documented in 216 cases (70.6%). The most common extragenital injury locations were the legs (138/306, 45.2%), upper arms (90/306, 29.4%), forearms (89/306, 29.1%), back (66/306, 21.6%), and face (65/306, 21.2%). The most frequent extragenital injury types were hematomas (184/306, 60.1%) and abrasions (119/306, 38.9%). Anogenital injuries were documented in 62 cases (20.3%). The most frequent anogenital injury locations were the anterior vagina/fornix (24/306, 7.8%), labia minora (14/306, 4.6%), and the labia majora, vestibulum, and anal region, each documented in 9 cases (2.9%). The most common anogenital injury types were lacerations (22/306, 7.2%), erythema (18/306, 5.9%), and abrasions (18/306, 5.9%).

**Table 1:** Complete demographic, assault-related, and injury characteristics of the study population (*n* = 306)

Characteristic	Value
Demographic and examination characteristics	
Age, years	29.96 ± 10.26 median 27.5 (22–36)
Height, cm	166.70 ± 7.85 median 166.5 (162–172)
Weight, kg	66.23 ± 17.39 median 62.0 (54–73.25)
BMI, kg/m²	23.79 ± 5.80 median 22.49 (19.77–25.96)
Time to examination, hours	38.46 ± 30.66 median 24 (24–48)
Affected individual and reported offender characteristics	
Sex of affected individual: female	288 (94.1)
Sex of affected individual: male	15 (4.9)
Sex of affected individual: diverse	3 (1.0)
Sex of reported offender: female	1 (0.3)
Sex of reported offender: male	298 (97.4)
Sex of reported offender: diverse	2 (0.7)
Sex of reported offender: unknown	5 (1.6)
Number of reported offenders: 1	257 (84.0)
Number of reported offenders: ≥ 2	38 (12.4)
Number of reported offenders: unknown	11 (3.6)
Reported perpetrator: current/former intimate partner	42 (13.7)
Reported perpetrator: known person	159 (52.0)
Reported perpetrator: unknown person	105 (34.3)
Reported alcohol involvement: no	155 (50.7)
Reported alcohol involvement: yes	150 (49.0)
Reported alcohol involvement: unknown/other	1 (0.3)
Reported drug involvement: no	229 (74.8)
Reported drug involvement: yes	51 (16.7)
Reported drug involvement: unknown	26 (8.5)
Disability of the victim	13 (4.2)
Self-inflicted injuries	45 (14.7)
Reported non-fatal strangulation	56 (18.3)
Reported sexual acts	
Any relevant oral, vaginal, or anal sexual act	256 (83.7)
Any vaginal act	226 (73.9)
Vaginal penile penetration	190 (62.1)
Vaginal digital penetration	85 (27.8)
Vaginal object penetration	10 (3.3)
Any anal act	67 (21.9)
Anal penile penetration	52 (17.0)
Anal digital penetration	19 (6.2)
Anal object penetration	3 (1.0)
Oral sexual violence	64 (20.9)
Unknown type of sexual violence	49 (16.0)
Number of reported sexual acts	
0	50 (16.3)
1	141 (46.1)
2	75 (24.5)
3	33 (10.8)
≥ 4	7 (2.3)
Extragenital injuries: anatomical location and type	
Any extragenital injury	216 (70.6)
Neck injury	58 (19.0)
Head injury	25 (8.2)
Facial injury	65 (21.2)
Back injury	66 (21.6)
Thoracic injury	55 (18.0)
Abdominal injury	16 (5.2)
Upper arm injury	90 (29.4)
Forearm injury	89 (29.1)
Hand injury	46 (15.0)
Leg injury	138 (45.2)
Petechiae	9 (2.9)
Abrasions	119 (38.9)
Hematomas	184 (60.1)
Bite marks	9 (2.9)
Lacerated wounds	5 (1.6)
Sharp-force injuries	2 (0.7)
Fractures	2 (0.7)
CIES severity scale: category 0	90 (29.4)
CIES severity scale: category 1	205 (67.0)
CIES severity scale: category 2	9 (2.9)
CIES severity scale: category 3	2 (0.7)
Forensic interpretation of injury patterns	
Blunt force by blows/kicks	144 (47.1)
Kissing/sucking injuries	38 (12.4)
Blunt force by objects	11 (3.6)
Defensive injuries	9 (2.9)
Fixation injuries	54 (17.6)
Strangulation marks	16 (5.2)
Undressing injuries	16 (5.2)
Spread injuries	19 (6.2)
Abutment injuries	33 (10.8)
Anogenital injuries: anatomical location and type	
Any anogenital injury	62 (20.3)
No anogenital injury	244 (79.7)
Labia majora injury	9 (2.9)
Labia minora injury	14 (4.6)
Clitoral injury	1 (0.3)
Vestibular injury	9 (2.9)
Posterior fourchette injury	8 (2.6)
Fossa navicularis injury	8 (2.6)
Hymenal injury	4 (1.3)
Anterior vagina/fornix injury	24 (7.8)
Perineal injury	8 (2.6)
Anal injury	9 (2.9)
Penile injury	1 (0.3)
Erythema	18 (5.9)
Edema	2 (0.7)
Hematoma	10 (3.3)
Abrasion	18 (5.9)
Laceration	22 (7.2)

Values are presented as mean ± standard deviation, median (interquartile range), or n (%). Percentages are based on the total cohort size unless otherwise indicated. Forensic interpretation categories represent forensic assessments of injury patterns and contextual information; they are presented descriptively and were not treated as independent anatomical injury outcomes

### Forensic interpretation categories of documented injury patterns

**Fig. 1:**
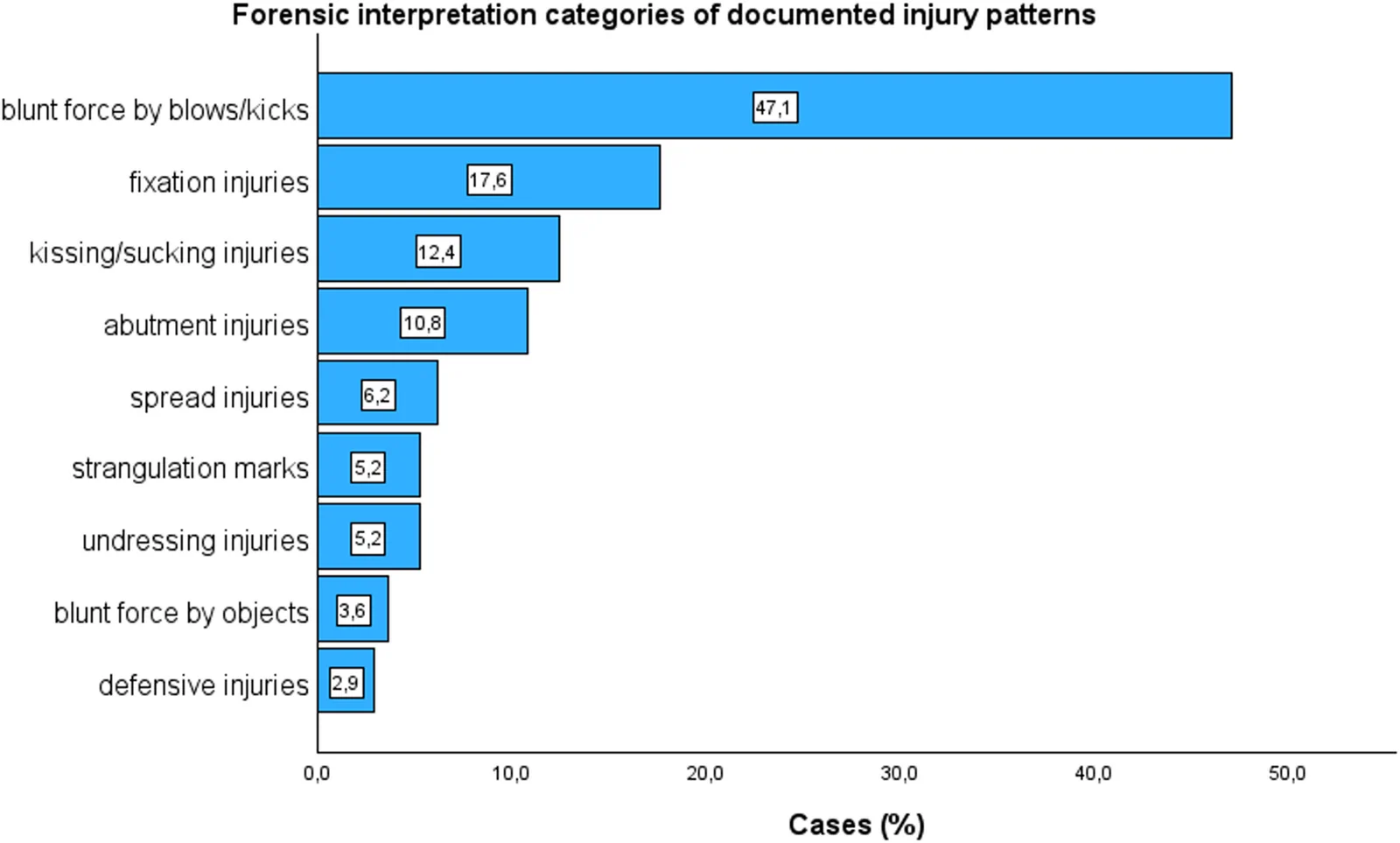
Forensic interpretation categories of documented injury patterns. Bars show the frequency of forensic interpretation categories assigned during medico-legal assessment of documented injury patterns in the total cohort (*n* = 306). Percentages are based on the total cohort size. Categories were not mutually exclusive and reflect forensic assessments based on injury morphology, localization, and contextual case information rather than independent anatomical injury outcomes. The recorded categories were blunt force by blows/kicks (144/306, 47.1%), fixation injuries (54/306, 17.6%), kissing/sucking injuries (38/306, 12.4%), abutment injuries (33/306, 10.8%), spread injuries (19/306, 6.2%), strangulation marks (16/306, 5.2%), undressing injuries (16/306, 5.2%), blunt force by objects (11/306, 3.6%), and defensive injuries (9/306, 2.9%)

Forensic interpretation categories were extracted from the finalized forensic examination reports in addition to anatomical injury locations and morphological injury types. These categories were not mutually exclusive and reflected the medico-legal interpretation documented in routine forensic practice, based on injury morphology, anatomical location, and contextual case information. They were analyzed descriptively and were not treated as independent anatomical injury outcomes. The most frequent forensic interpretation category was blunt force by blows or kicks, documented in 144/306 cases (47.1%). Fixation injuries were recorded in 54 cases (17.6%), kissing or sucking injuries in 38 cases (12.4%), and abutment injuries in 33 cases (10.8%). Less frequent categories included spread injuries (19/306, 6.2%), strangulation marks and undressing injuries, each documented in 16 cases (5.2%), blunt force by objects (11/306, 3.6%), and defensive injuries (9/306, 2.9%). The distribution of forensic interpretation categories is shown in Fig. 1.

### Anatomical correspondence between reported sexual acts and documented injuries

**Fig. 2:**
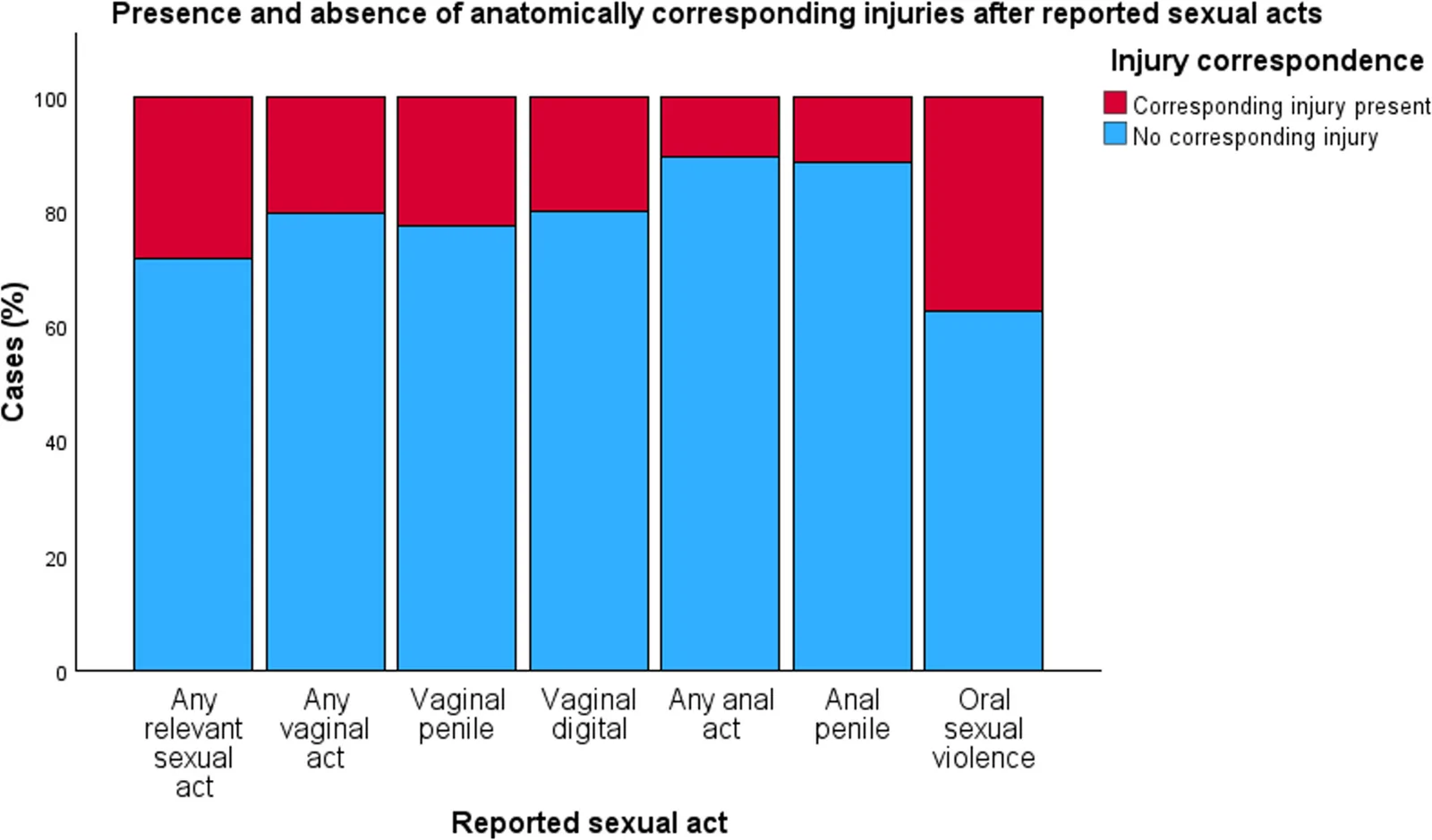
Presence and absence of anatomically corresponding injuries after reported sexual acts. Stacked bars show the proportion of cases with and without predefined anatomically or regionally corresponding visible injuries among cases reporting the respective sexual act. Corresponding visible injuries were documented in 72/256 cases (28.1%, 95% CI 23.0–33.9) with at least one reported oral, vaginal, or anal sexual act. Detection rates were 46/226 (20.4%, 95% CI 15.6–26.1; *p* = 0.946) for any vaginal act with any anogenital injury, 43/190 (22.6%, 95% CI 17.3–29.1; *p* = 0.187) for penile-vaginal penetration with any anogenital injury, 17/85 (20.0%, 95% CI 12.9–29.7; *p* = 0.944) for digital-vaginal penetration with any anogenital injury, 7/67 (10.4%, 95% CI 5.2–20.0; *p* < 0.001) for any anal act with anal injury, 6/52 (11.5%, 95% CI 5.4–23.0; *p* < 0.001) for penile-anal penetration with anal injury, and 24/64 (37.5%, 95% CI 26.7–49.7; *p* < 0.001) for oral sexual violence with facial injury. P-values refer to chi-square tests or Fisher’s exact tests, as appropriate, comparing injury prevalence between cases with and without the respective reported sexual act. No p-value is shown for the composite endpoint because this analysis was restricted to cases with at least one reported oral, vaginal, or anal sexual act. “Any anogenital injury” corresponds to the composite anogenital injury variable

Anatomical correspondence between reported sexual acts and documented visible injuries was assessed in cases with reported oral, vaginal, or anal sexual acts. Because reported sexual acts were not mutually exclusive, individuals could contribute to more than one modality-specific analysis. Among the 256 cases with at least one reported oral, vaginal, or anal sexual act, at least one predefined anatomically or regionally corresponding visible injury was documented in 72 cases (28.1%, 95% CI 23.0–33.9). Conversely, 184 cases (71.9%) showed no corresponding visible injury. This overall pattern is illustrated in Fig. 2 and detailed in Supplementary Table S1. For vaginal acts, corresponding anogenital injuries were uncommon. Among cases reporting any vaginal act, anogenital injuries were documented in 46/226 cases (20.4%, 95% CI 15.6–26.1), with no significant association between reported vaginal acts and the presence of anogenital injury (*p* = 0.946). Similarly, anogenital injuries were documented in 43/190 cases (22.6%, 95% CI 17.3–29.1) after reported penile-vaginal penetration (*p* = 0.187) and in 17/85 cases (20.0%, 95% CI 12.9–29.7) after reported digital-vaginal penetration (*p* = 0.944). For anal acts, documented anal injuries were infrequent in absolute terms but were significantly associated with reported anal sexual violence. Anal injuries were present in 7/67 cases (10.4%, 95% CI 5.2–20.0) reporting any anal act (*p* < 0.001) and in 6/52 cases (11.5%, 95% CI 5.4–23.0) reporting penile-anal penetration (*p* < 0.001). Despite these significant associations, corresponding anal injuries were absent in the majority of cases reporting anal acts. Oral sexual violence was significantly associated with documented facial injuries, which were predefined as regionally corresponding visible injuries. Facial injuries were present in 24/64 cases (37.5%, 95% CI 26.7–49.7) reporting oral sexual violence (*p* < 0.001), whereas 40/64 cases (62.5%) showed no corresponding facial injury. Overall, predefined corresponding visible injuries were less frequent than absent corresponding findings across all reported sexual act categories.

### Factors associated with absent anatomically corresponding injuries

**Fig. 3:**
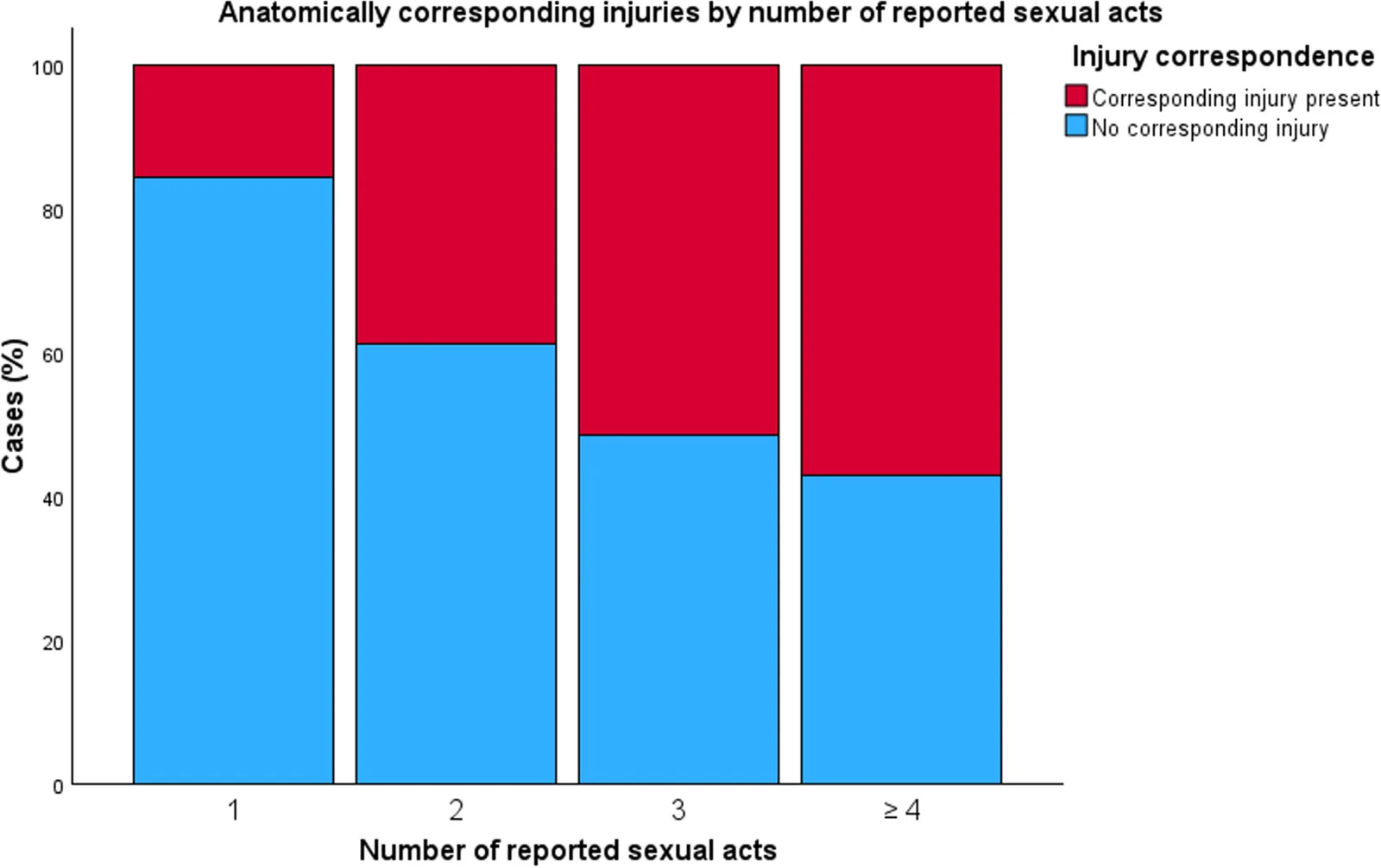
Anatomically corresponding injuries by number of reported sexual acts. Stacked bars show the proportion of cases with and without at least one predefined anatomically or regionally corresponding visible injury according to the number of reported oral, vaginal, or anal sexual acts. The analysis was restricted to cases with at least one reported relevant sexual act (*n* = 256). The proportion of cases with at least one corresponding injury increased from 22/141 (15.6%) among cases reporting one sexual act to 29/75 (38.7%) among cases reporting two acts, 17/33 (51.5%) among cases reporting three acts, and 4/7 (57.1%) among cases reporting four or more acts. Categories with four, five, and seven reported acts were combined because of small case numbers. The number of reported sexual acts differed significantly between cases with and without corresponding visible injuries in Mann–Whitney U testing and was the only independently associated variable in multivariable logistic regression

Among cases with at least one reported oral, vaginal, or anal sexual act, the absence of predefined corresponding visible injuries was further evaluated in relation to demographic and assault-related variables. No significant association was observed between absent corresponding injury and age, time to examination, reported alcohol involvement, reported drug involvement, number of reported offenders, or reported offender category. In contrast, the number of reported sexual acts differed significantly between cases with and without corresponding visible injuries. The proportion of cases with at least one corresponding injury increased with the number of reported sexual acts: corresponding injuries were documented in 22/141 cases (15.6%) reporting one sexual act, 29/75 cases (38.7%) reporting two acts, 17/33 cases (51.5%) reporting three acts, and 4/7 cases (57.1%) reporting four or more acts (Fig. 3). Categories with four, five, and seven reported acts were combined because of small case numbers. In multivariable binary logistic regression including age, time to examination, reported alcohol involvement, reported drug involvement, and number of reported sexual acts, the overall model was statistically significant (χ² = 28.922, df = 6, *p* < 0.001; Nagelkerke R² = 0.154). The number of reported sexual acts was the only independent variable associated with absence of corresponding visible injury (OR = 0.440, 95% CI 0.309–0.625, *p* < 0.001), indicating lower odds of absent corresponding injury with increasing numbers of reported sexual acts. Age, time to examination, reported alcohol involvement, and reported drug involvement were not independently associated with the outcome. Full univariable and multivariable results are provided in Supplementary Table S2.

## Discussion

The present study analyzed visible genital, anogenital, and extragenital injury patterns in 306 adults examined after reported sexual assault in a forensic outpatient setting. The main finding was that predefined anatomically or regionally corresponding visible injuries were absent in the majority of cases with reported oral, vaginal, or anal sexual acts. Among 256 individuals reporting at least one such act, only 28.1% had at least one corresponding visible injury, whereas 71.9% showed no corresponding finding. This supports the view that the absence of visible injury is common after reported sexual assault and should not be interpreted as evidence against the reported event. Extragenital injuries were considerably more frequent than anogenital injuries. In the total cohort, extragenital injuries were documented in 70.6% of cases, whereas anogenital injuries were documented in 20.3%. This pattern is consistent with previous studies showing wide variability in physical findings after sexual assault and a frequent absence of genital or anal injuries despite reported penetrative contact [6–16]. The relatively low proportion of anogenital injuries in the present cohort further supports the view that genital findings have limited diagnostic value when interpreted in isolation. This is particularly relevant because previous studies comparing consensual and non-consensual intercourse have demonstrated substantial overlap in genital injury patterns [4, 5]. In particular, erythema should be interpreted cautiously as it is a non-specific finding and does not necessarily indicate traumatic injury. The analysis of anatomical correspondence adds a further level of interpretation. For vaginal acts, corresponding anogenital injuries were uncommon and were not significantly associated with reported vaginal acts. Anogenital injuries were documented in only 20.4% of cases reporting any vaginal act and in 22.6% of cases reporting penile-vaginal penetration. These findings underline that the absence of genital or anogenital injury is not unusual, even when vaginal penetration is reported. From a forensic perspective, this is important because negative findings must be communicated carefully: they do not exclude sexual assault, but rather reflect the limited sensitivity of visible injury documentation. In contrast, reported anal sexual violence was significantly associated with documented anal injury. However, the absolute detection rate remained low: anal injuries were present in only 10.4% of cases reporting any anal act and in 11.5% of cases reporting penile-anal penetration. This distinction between statistical association and practical sensitivity is essential. A significant association indicates that anal injuries were more frequent among individuals reporting anal acts than among those who did not, but most individuals reporting anal acts still had no visible anal injury. Therefore, even anatomically plausible associations should not be overinterpreted as reliable indicators at the individual case level.

Oral sexual violence was significantly associated with documented facial injuries. However, this finding requires cautious interpretation because facial injuries were predefined as regionally corresponding rather than anatomically specific. Facial injuries may occur in many contexts, including blows, falls, restraint, or other forms of physical violence, and are not specific for oral sexual violence. Accordingly, the observed association should be understood as a regional correspondence within the predefined analytical framework, not as proof of a specific mechanism of injury production. The number of reported sexual acts was the only variable independently associated with the absence of corresponding visible injury in the multivariable model. Increasing numbers of reported sexual acts were associated with lower odds of absent corresponding injury. This finding is plausible but should be interpreted cautiously. Multiple reported acts increase the number of anatomical regions potentially exposed to injury and therefore increase the probability of detecting at least one corresponding visible finding. Thus, the association may partly reflect the structure of the composite endpoint rather than a direct relationship between assault severity and injury formation. The forensic interpretation categories extracted from the finalized examination reports provide additional descriptive information on how injury patterns were assessed in routine medico-legal practice. The most frequent category was blunt force by blows or kicks, followed by fixation injuries, kissing or sucking injuries, and abutment injuries. These categories should not be understood as independent anatomical injury outcomes or as objective reconstruction of the assault mechanism. Rather, they reflect the medico-legal interpretation of injury morphology, location, and contextual information documented in the original forensic reports. Their value lies in describing how documented findings are interpreted in forensic practice, while their evidentiary meaning remains dependent on the full case context. Methodologically, the present study has several strengths. It is based on a comparatively large forensic outpatient cohort collected over an eight-year period, and all cases were examined in a specialized clinical forensic setting. The inclusion of individuals irrespective of visible injury status reduces the risk of selecting only injury-positive cases and allows a more realistic assessment of the frequency of absent findings. In addition, the study distinguishes between anatomical injury location, injury morphology, reported sexual acts, and forensic interpretation categories, thereby avoiding a direct equation of injury presence with assault mechanism. Several limitations must also be considered. First, the study was retrospective and based on finalized routine forensic examination reports. Although these reports were generated in a specialized forensic setting and supported by photographic documentation where available, no independent blinded re-assessment of all injuries was performed for the purpose of this study. Therefore, the analysis reflects documented routine forensic practice rather than a prospectively standardized research examination. Second, reported sexual acts were based on the affected individuals’ accounts as documented at the time of examination. In cases of intoxication, impaired memory, delayed reporting, or psychological distress, the exact assault dynamics may have been unknown or uncertain. This is reflected by the subgroup with unknown or uncertain sexual dynamics. Such uncertainty is inherent to real-world forensic assessment after sexual assault but limits the precision of act-specific analyses. Third, the definition of anatomical correspondence was operational and predefined for the purpose of this study. For vaginal acts, any anogenital injury was considered corresponding; for anal acts, anal injury was considered corresponding; and for oral sexual violence, facial injury was considered regionally corresponding. These definitions allowed systematic analysis but are imperfect. In particular, facial injuries are not specific to oral sexual violence, and anogenital injuries may occur outside the direct context of vaginal penetration. The findings should therefore be interpreted as predefined anatomical or regional correspondence rather than definitive causal attribution. Fourth, another limitation concerns the inclusion of erythema as an anogenital injury. Erythema is a non-specific finding and its forensic significance remains debated, as it may result from physiological variation, irritation, or examination-related factors rather than trauma. Consequently, the inclusion of erythema may have led to a slight overestimation of the prevalence of anogenital injuries in the present cohort. Fifth, several reported sexual act categories were not mutually exclusive. Individuals could contribute to more than one modality-specific analysis, which reflects the complexity of sexual assault but also limits the independence of subgroup comparisons. Some subgroups, particularly object-related penetration and male or diverse individuals, were small, limiting meaningful subgroup-specific interpretation. Moreover, no correction for multiple testing was applied because the analysis was exploratory. Sixth, the study focused on visible injuries documented during forensic examination. It therefore does not capture transient injuries that may have resolved before examination, injuries not visible externally, or findings that would require additional diagnostic procedures. The lack of association between time to examination and absence of corresponding visible injury in the present analysis should not be interpreted as evidence that timing is irrelevant. Rather, the composite endpoint used here combines different injury types and anatomical regions with different visibility windows. The time-dependent nature of visible findings is also evident after violence against the neck: in a previous analysis from the same forensic outpatient setting, petechiae were documented in only 10% of non-fatal strangulation cases, and their detectability decreased with increasing time to examination [17]. Therefore, early examination and careful documentation remain essential. Finally, the data originate from a single urban forensic outpatient clinic with established procedures for clinical forensic examination. The results may not be directly generalizable to settings with different referral pathways, examination protocols, documentation standards, or patient populations. At the same time, the cohort reflects real-world forensic practice, including cases with unclear dynamics, absent visible injuries, and mixed patterns of sexual and physical violence.

Overall, the findings demonstrate that visible injuries after reported sexual assault are frequent at extragenital sites but much less common in anatomically corresponding anogenital or anal locations. Even when statistically significant associations were observed, particularly for anal acts and oral sexual violence, corresponding visible injuries were absent in the majority of cases. These results support a cautious and contextual forensic interpretation: the presence of a corresponding injury may support the assessment of consistency with the reported history, but the absence of such injury must not be used to exclude reported sexual assault.

## Conclusion

Visible injuries after reported sexual assault were common at extragenital sites but were absent in anatomically or regionally corresponding locations in most cases reporting oral, vaginal, or anal sexual acts. Even where significant associations were observed, particularly for anal acts and oral sexual violence, corresponding visible injuries were documented only in a minority of cases. The findings therefore reinforce a central principle of forensic assessment: the presence of a corresponding injury may support consistency with the reported history, but the absence of visible injury must not be interpreted as excluding the reported assault. Structured forensic documentation and cautious contextual interpretation are essential. 

## Supplementary Information

Supplementary Material 1. Table S1: Anatomical correspondence between reported sexual acts and documented injuries. Table S2: Univariable and multivariable analyses of factors associated with absent anatomically corresponding injury. Table S3: Clinical Injury Extent Score used for grading extragenital injury severity.

## Data Availability

The individual-level data analyzed in this study are not publicly available and cannot be shared by the authors due to the sensitive nature of forensic case documentation, medical confidentiality, and applicable data protection restrictions. Aggregated data supporting the main findings are included in this article and its supplementary material.
