# Supplementary material for: Visible injury patterns in relation to reported sexual acts after sexual assault: a retrospective cohort study
Source: Int J Legal Med. 2026 Aug 1;140(6):4265–76. doi: 10.1007/s00414-026-03941-8 (PMC13658310; doi:10.1007/s00414-026-03941-8)
Supplement: Supplementary file 1 [file 414_2026_3941_MOESM1_ESM.docx]

Supplementary material 
Supplementary table S1 Anatomical correspondence between reported sexual acts and documented injuries
Reported sexual act
Corresponding documented injury
Reported act, n
Corresponding injury present n (%)
95% CI
Corresponding injury absent n (%)
p-value
Any relevant oral, vaginal, or anal sexual act
Any anatomically or regionally corresponding injury
256
72 (28.1)
23.0–33.9
184 (71.9)
—
Any vaginal act
Any anogenital injury
226
46 (20.4)
15.6–26.1
180 (79.6)
.946
Vaginal penile penetration
Any anogenital injury
190
43 (22.6)
17.3–29.1
147 (77.4)
.187
Vaginal digital penetration
Any anogenital injury
85
17 (20.0)
12.9–29.7
68 (80.0)
.944
Any anal act
Anal injury
67
7 (10.4)
5.2–20.0
60 (89.6)
<.001
Anal penile penetration
Anal injury
52
6 (11.5)
5.4–23.0
46 (88.5)
<.001
Oral sexual violence
Facial injury
64
24 (37.5)
26.7–49.7
40 (62.5)
<.001
P-values refer to chi-square tests or Fisher’s exact tests, as appropriate, comparing injury prevalence between cases with and without the respective reported sexual act. For the overall composite endpoint, no p-value is shown because the analysis is restricted to cases with at least one relevant reported oral, vaginal, or anal sexual act.

Supplementary table S2 Univariable and multivariable analyses of factors associated with absent anatomically corresponding injury
A. Univariable analyses
Variable
Category / comparison
No corresponding injury, n/N (%) or direction of effect
Test statistic
p-value
Reported alcohol involvement
No
108/147 (73.5)
χ² = 0.434
.510

Yes
76/109 (69.7)


Reported drug involvement
No
143/205 (69.8)
χ² = 2.676
.262

Yes
33/42 (78.6)


Unknown to the affected individual
8/9 (88.9)


Number of reported offenders
1
163/224 (72.8)
χ² = 0.964
.617

2 or >2
20/30 (66.7)


Unknown
1/2 (50.0)


Relationship to the reported Perpetrator 
Current/former intimate partner
29/42 (69.0)
χ² = 1.228
.541

Known person
101/135 (74.8)


Unknown person
54/79 (68.4)


Age
No significant rank difference
—
Mann–Whitney U = 6385.5
.654
Time to examination
No significant rank difference
—
Mann–Whitney U = 6375.5
.632
Number of reported sexual acts
Lower in cases with no corresponding injury
—
Mann–Whitney U = 4148.5
<.001

B. Multivariable logistic regression
Predictor
OR
95% CI
p-value
Age
1.006
0.976–1.037
.710
Time to examination
1.003
0.993–1.013
.575
Reported alcohol involvement
0.690
0.376–1.265
.230
Reported drug involvement: yes
1.690
0.722–3.955
.227
Reported drug involvement: unknown to the affected individual
3.669
0.421–31.967
.239
Number of reported sexual acts
0.440
0.309–0.625
<.001
Analyses were restricted to cases with at least one reported oral, vaginal, or anal sexual act (n = 256). The dependent variable was absence of anatomically corresponding injury. Categorical variables were analysed using chi-square tests; continuous or ordinal variables were analysed using Mann–Whitney U tests. Drug involvement was entered into the multivariable model as a categorical predictor with “no drug involvement” as the reference category. Model fit: χ² = 28.922, df = 6, p < .001; Nagelkerke R² = 0.154; Hosmer–Lemeshow p = .157.

Supplementary table S3 Clinical Injury Extent Score used for grading extragenital injury severity
CIES category
Severity category
Operational definition
0
No injury
No documented visible extragenital injury.
1
Mild injury
Minor injuries without expected impact on physical function and not requiring medical treatment.
2
Moderate injury
Injuries expected to affect physical function and/or requiring medical treatment.
3
Severe injury
Major injury, such as fracture, internal organ injury, head injury with concussion, attempted strangulation with clinically relevant findings, or injury requiring high-dependency/intensive care.
